# SARS-CoV-2 ORF8: A Rapidly Evolving Immune and Viral Modulator in COVID-19

**DOI:** 10.3390/v15040871

**Published:** 2023-03-29

**Authors:** Ariana Arduini, Frederique Laprise, Chen Liang

**Affiliations:** 1 Lady Davis Institute, Jewish General Hospital, Montreal, QC H3T 1E2, Canada; ariana.arduini@mail.mcgill.ca (A.A.); frederique.laprise@mail.mcgill.ca (F.L.); 2 Department of Medicine, McGill University, Montreal, QC H3G 2M1, Canada; 3 Department of Microbiology and Immunology, McGill University, Montreal, QC H3A 2B4, Canada

**Keywords:** SARS-CoV-2, COVID-19, ORF8, accessory protein, immune evasion, viral mimicry

## Abstract

The COVID-19 pandemic has resulted in upwards of 6.8 million deaths over the past three years, and the frequent emergence of variants continues to strain global health. Although vaccines have greatly helped mitigate disease severity, SARS-CoV-2 is likely to remain endemic, making it critical to understand its viral mechanisms contributing to pathogenesis and discover new antiviral therapeutics. To efficiently infect, this virus uses a diverse set of strategies to evade host immunity, accounting for its high pathogenicity and rapid spread throughout the COVID-19 pandemic. Behind some of these critical host evasion strategies is the accessory protein Open Reading Frame 8 (ORF8), which has gained recognition in SARS-CoV-2 pathogenesis due to its hypervariability, secretory property, and unique structure. This review discusses the current knowledge on SARS-CoV-2 ORF8 and proposes actualized functional models describing its pivotal roles in both viral replication and immune evasion. A better understanding of ORF8’s interactions with host and viral factors is expected to reveal essential pathogenic strategies utilized by SARS-CoV-2 and inspire the development of novel therapeutics to improve COVID-19 disease outcomes.

## 1. Introduction

Severe acute respiratory syndrome coronavirus 2 (SARS-CoV-2) is the causative agent for the coronavirus disease 2019 (COVID-19) pandemic, which surfaced in late 2019 and remains a global threat [1]. While largely manifesting as a mild upper respiratory tract infection, a significant proportion of patients show signs of potentially fatal lower respiratory illness, acute respiratory distress syndrome (ARDS), and multi-organ failure [2,3]. SARS-CoV-2 is the third most highly pathogenic member of the *Coronaviridae* family and genus Betacoronavirus to emerge in the last two decades, after SARS-CoV and Middle East respiratory syndrome CoV (MERS-CoV), with which it shares a 79% and 50% sequence identity, respectively [4]. The SARS-CoV-2 genome (positive single-stranded RNA, ~30 kb) contains multiple open reading frames (ORFs) encoding twenty-nine viral proteins. These include sixteen nonstructural genes (Nsp1-16) which are encoded by ORF1a and ORF1b to mediate viral RNA replication and transcription, four structural genes (spike (S), envelope (E), nucleocapsid (N), and membrane (M)) which form the virus particles, and nine accessory genes (ORF3a, 3b, 6, 7a, 7b, 8, 9a, 9b, and 10), which are non-essential for viral replication yet are critical in mitigating host–virus interactions [5].

Among these nine viral accessory factors, SARS-CoV-2 ORF8 is particularly notable. Being a secreted viral protein and having the largest protein interactome network of all the accessory factors, ORF8 has an array of functions which altogether antagonize host pathways to promote SARS-CoV-2 pathogenesis [6]. ORF8 specifically promotes both intracellular and systemic changes which contribute to the formation of an environment supportive of viral replication to be shielded from host immunity. These changes include the induction of endoplasmic reticulum (ER) stress, activation of unfolded protein responses (UPR), alteration of gene expression through histone mimicry, antagonization of multiple immune signaling pathways (major histocompatibility complex (MHC-I), type-I interferon (IFN), and interleukin 17 (IL-17)), and alteration of monocyte function [7,8,9,10,11]. ORF8 is also remarkable for its hypervariability and unique structural properties, namely, its immunoglobulin (Ig) domain and ability to dimerize [12,13]. The important role of SARS-CoV-2 ORF8 is further emphasized in patients experiencing milder COVID-19 disease when infected with a 382-nucleotide deletion of ORF8 [14].

Understanding the role of ORF8 is therefore critical in elucidating the mechanisms of SARS-CoV-2 disease, and this role has been the subject of several prior reviews [15,16,17,18]. The present work will further consider our current knowledge on this protein, focusing on its novel structural properties and hypervariability over the course of the COVID-19 pandemic. We also aim to highlight ORF8 as an influential player in SARS-CoV-2 pathogenesis through its interactions with different cellular pathways and host immune responses. 

## 2. ORF8 Is a Highly Immunogenic, Secreted Viral Protein Shared by Sarbecoviruses

**Figure 1:**
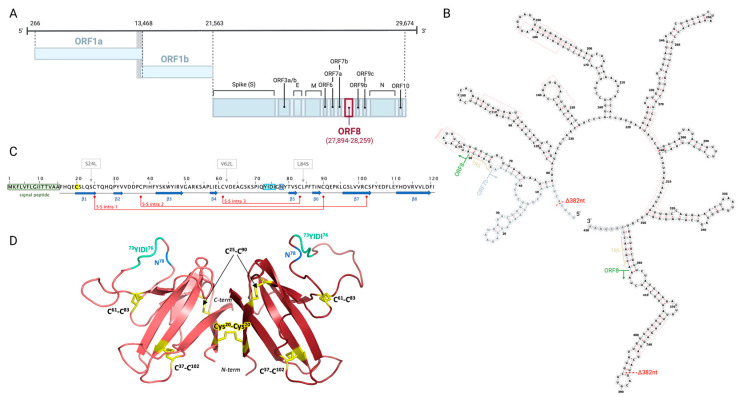
Structure of SARS-CoV-2 *ORF8* gene and encoded ORF8 protein. (**A**) *ORF8* location within the SARS-CoV-2 genome (NCBI reference NC_045512.2, Gene ID 43740577), with nucleotide positions indicated. Created in BioRender.com, accessed on 9 March 2023 (**B**) *ORF8* RNA secondary structure adapted from Pereira, F. [20], generated in ViennaRNA Web Services. The coding region of *ORF8* is indicated. Also shown is the Δ382 deletion. (**C**) ORF8 amino acid sequence. The N-terminal signal peptide, dimerization residue C20, 73YIDI76 motif, and glycosylation residue N78 are highlighted in green, yellow, cyan, and blue, respectively. β-sheets are represented by blue arrows. Intramolecular disulfide bridges are shown in maroon. Three frequently emerging mutations S24L, V62L, and L84S are shown. (**D**) Cartoon representation of the dimerized SARS-CoV-2 ORF8 crystal structure (PDB: 7JTL) generated in PyMOL 2.5.4. Disulfide bridges are highlighted in yellow, 73YIDI76 is highlighted in cyan, and N78 is highlighted in blue. TRS; transcription regulatory sequence.

The genomic organization of SARS-CoV-2 and other *Coronaviridae* family members is shared for structural and non-structural genes. However, their accessory genes vary in number, location, and similarity [19]. Within the accessory genes, *ORF8* is poorly conserved and has been associated with SARS-CoV-2 virulence [20]. The *ORF8* gene has only been detected in Sarbecoviruses, most of which retain a single continuous ORF8 protein [20,21,22]. In SARS-CoV-2, *ORF8* spans 366 nucleotides of the viral genome (positions 27,894–28,259), following the *M* and preceding the *N* gene in a cluster also including *ORF6*, *ORF7a*, and *ORF7b* (NCBI reference NC_045512.2, Gene ID 43740577) (Figure 1A,B). It encodes for a 121-amino acid (aa) protein (Protein ID YP_009724396.1) with an N-terminal hydrophobic signal peptide (1–15 aa) and an ORF8 chain (16–121 aa) (Figure 1C,D) [23]. 

There are several theories on the origin of SARS-CoV-2 ORF8. Its gene is found within a recombination hotspot in SARS-CoV-2 and SARS-CoV and in bat and pangolin SARS related-CoVs (SARSr-CoVs) [24]. Despite the functional similarities between SARS-CoV-2 ORF8 and SARS-CoV ORF8, the two share only 55.4% nucleotide and 30% amino acid similarity [23]. Even compared to the bat-CoV RaTG13, thought to be the closest relative of SARS-CoV-2, *ORF8* shares 93% nucleotide similarity [23]. Whether it may have arisen from bat-CoV RaTG13 *ORF8* or from the differentiation of a fragmented *ORF8ab* sequence from SARS-CoV is not clear [25,26,27]. Moreover, *ORF8* and *ORF7a* were believed to have originated from a gene duplication event due to extensive structural similarities between the two proteins, including the presence of a 15-residue signal peptide, the alignment of an Ig-like domain, and their loss of expression in the Gamma/Deltacoronavirus genera and MERS-CoV clade [16,20,28,29]. However, the low homology between the *ORF8* and *ORF7a* genes (45%) and encoded proteins (18%) suggests their divergence through independent evolutionary events [16,20,28,29]. *ORF7a* is also more conserved between bat SARSr-CoVs, and lacks interactions with known ORF8-binding proteins despite structural similarities with ORF8 [29]. The ORF8 of SARS-CoV-2 is thus unique, and its greater tendency to mutate suggests its role in assisting virus adaptation to new hosts.

Along with its hypervariability, the SARS-CoV-2 ORF8 protein stands out for its novel capacity to dimerize compared to its counterparts in other Sarbecoviruses. Monomeric units of this protein are comprised of an α-helix, followed by a β-sandwich of seven β-strands, stabilized through three intramolecular disulfide bonds (C25-C90, C37-C102, C61-C83) (Figure 1C,D) [13,15,30]. Dimerization of the two monomers is then mediated by intermolecular disulfide bonding between C20 residues, and is strengthened by four intermolecular salt bridges (D119-R115, R115-E92) and multiple intermolecular hydrogen bonds (F120-K53, K53-S24, Q18-L22, R52-I121), revealed to be co-acting in dimer stabilization through in silico analysis [13,28,30,31]. The disulfide bond at C20 also involves a 73YIDI76 motif unique to SARS-CoV-2 ORF8, further stabilizing the non-covalent dimer interface (Figure 1C,D) [28,30]. Given that both the C20 and 73YIDI76 motif are absent from SARS-CoV and SARSr-CoV ORF8 proteins, these are expected to promote higher-order assembly of SARS-CoV-2 ORF8 and enhance interactions with host factors, thus contributing to viral pathogenesis [13,28]. 

Additionally, of particular interest is the Ig domain within the SARS-CoV-2 ORF8 β-sandwich, theorized to mimic host immune molecules and counter host immune pressure on the virus (Figure 1C) [13,32]. Ig domain expression by viruses is known to be a strategy in modulating host immunity, mimicking host functions and acting as a molecular trap [13,16,32]. While Ig domains are also distinctively found among Betacoronaviruses ORF8 and ORF7a proteins, as well as Alphacoronavirus ORF9 and ORF10 proteins, the Ig domain in SARS-CoV-2 ORF8 is distinct from its homologues due to its lack of a C-terminal transmembrane domain and the presence of a long insertion between strands 3 and 4 (residues 46 to 83) of the β-sandwich core, which is expected to facilitate dimerization due to the novel addition of a conserved cysteine [16,32].

A unique property of ORF8 that is not shared by other SARS-CoV-2 proteins is its ability to be secreted from infected cells. In both COVID-19 patient serum and cell culture supernatants, SARS-CoV-2 ORF8 is detected as a glycoprotein homodimer, with its levels positively correlating to both disease course and mortality [33]. ORF8 secretion depends on its signal peptide as well as N-linked glycosylation on N78, and mutagenesis of N78 dramatically reduces ORF8 secretion in vitro [34,35]. Further study suggests that signal-peptide-deficient ORF8 is unglycosylated and secreted via an unconventional pathway, whereby it may additionally contribute to COVID-19 cytokine storm [35]. However, the wild-type ORF8 may not preferably adopt the unconventional pathway for secretion, since the vast majority of ORF8 in the sera of COVID-19 patients and in the supernatant of SARS-CoV-2 infected cells is glycosylated [36]. SARS-CoV ORF8 has also been experimentally shown to be similarly secreted and glycosylated on N81, but it is hypothesized to secrete via different pathways [35]. Interestingly, ORF8 is released from SARS-CoV-2 infected cells as a homodimer, yet the intermolecular disulfide bonding at C20 is dispensable for this property [30,34,37]. 

The prominence of secreted ORF8 in COVID-19 is further highlighted by this protein’s high immunogenicity at both early and late stages of disease. Along with S, N, and ORF3b, ORF8 elicits strong and specific antibody responses, with its most immunodominant epitopes being the N-terminal α-helix, residues spanning the β2 and β3 sheets, and the loop between the β4 and β5 sheets [38,39,40]. Notably, anti-ORF8 IgG responses are extremely durable over time in convalescent plasma, being detected 100 days post-symptom onset [38]. Intriguingly, SARS-CoV-2 ORF8 shows one of the lowest homologies to SARS-CoV among all viral proteins, potentially leading to differences in the humoral response to these two Sarbecoviruses [38]. While these anti-ORF8 antibodies may not confer protection, the robustness and stability of anti-ORF8 responses may make them useful markers for improving COVID-19 diagnostics during acute and late infection, thus meriting further consideration [39,41].

## 3. ORF8 Evolution during the COVID-19 Pandemic

ORF8 is one of the most hypervariable regions in SARS-CoV-2 after the S protein receptor-binding domain (RBD), and presents the highest mutation density among nonstructural proteins [42]. On one hand, this can be associated with genomic instability at the *ORF8* gene. Several large RNA hairpins have been predicted in the *ORF8* RNA secondary structure as a result of perfect nucleotide repeats, and polymorphic positions are more common within these paired RNA structures (Figure 1B) [20]. Given that these structures frequently exhibit genomic instability and are known to have regulatory functions in coronavirus translation and replication, their contribution to ORF8 evolution needs further investigation [20,43]. Indeed, the mutational profile of *ORF8* over the last three years of the COVID-19 pandemic suggests strong selective pressure for such rapid evolution.

Early in the COVID-19 pandemic, the divergence of three major phylogenetic clades was detected, the largest being characterized by a single nucleotide polymorphism (SNP) in *ORF8* at position 28,144 (251T>C, L84S) [42,44]. This nonsynonymous mutation of leucine to serine, which was significantly linked to another SNP at position 8782 (*ORF1ab*: T8517C, synonymous), defined the deadly “L” and less virulent “S” lineages of SARS-CoV-2 [44,45]. While the L84S mutation is thought to hinder non-covalent dimer interactions in ORF8 mediated by the 73YIDI76 motif, it is also positioned in a predicted peptide-ligand binding groove in the Ig domain with a potential role in host–virus interaction [13,32]. Correspondingly, this variant has been associated with attenuated inflammation in vitro [23,45,46]. Although the combinatory effects of the L84S and *ORF1ab* mutations have not been described, it is suspected that L84S arose from selective pressure that led to decreased viral pathogenesis or enhanced adaptability in human hosts [22]. In contrast to amino acid 84 being the most variable site in ORF8, with S24L and V62L variants also frequently emerging, other sites including dimerization residue C20 and glycosylation residue N78 have been highly conserved, suggesting their central role in ORF8 function [22,32,47,48]. Despite some sites being more polymorphic than others, the mutational distribution of SARS-CoV-2 *ORF8* remains widespread. Mutations have been detected in both the ORF8 chain and its signal peptide, with an estimated 0.223 mutations/aa site overall, many of which are predicted to affect the structural stability of the protein [23,42]. 

Several deletions affecting the amino acid length of ORF8 have also been described [21,45]. A well-characterized example is the 382-nucleotide deletion (Δ382 *ORF8*) of positions 27,848 to 28,229, which removes the C-terminal end of *ORF7b* and 336 nucleotides (91.8%) of *ORF8*, creating a hybrid ORF7b that is seven amino acids shorter than normal and is fused to five putatively translated C-terminal ORF8 residues (T-F-V-L-F) (Figure 1B) [16,20,49]. Notably, patients infected with the Δ382 *ORF8* variant experience less severe COVID-19 disease, displaying milder symptoms with later onset, less systemic inflammation, and a reduced risk of hypoxia development compared to wild-type virus [50,51,52]. Further suggesting their importance in altering SARS-CoV-2 virulence, *ORF8* deletion variants often occur with at least one relevant S protein mutation allowing for better viral attachment or immune evasion [52]. This is especially apparent in more pathogenic SARS-CoV-2 variants of concern (VOCs), such as Alpha (B.1.1.7) containing a Q27stop, and the more transmissible variant of concern Delta (B.1.617.2) holding a del119/120 (D119-F) predicted in silico to destabilize the ORF8 dimer through loss of three salt bridges and one hydrogen bond [31,52,53]. Viral strains lacking a complete *ORF8* have also been reported in immunocompromised patients chronically infected with SARS-CoV-2, and such strains often do not efficiently suppress immune responses and produce milder, but prolonged infections with more opportunities for transmission [49,52]. Interestingly, an *ORF8*-deficient variant with a Q18stop and several associated S protein mutations was detected in a lymphoma patient with SARS-CoV-2 infection, suggesting that selective pressures to maintain ORF8 in such patients are lost [52,54]. 

Consistently, partial or complete deletions of the *ORF8* genomic region also occurred during the 2003 SARS-CoV epidemic, which was likely important for human adaptation and transmission [55,56]. One of the most remarkable changes observed in genomic isolates of SARS-CoV shortly after zoonotic transmission from civets was the acquisition of a 29-nucleotide deletion in *ORF8*, splitting it into two *ORFs*. Instead of the wild-type ORF8 (122aa) present in early viral isolates, later coined ORF8ab, this split produced two proteins with elusive functions, namely, ORF8a (39aa) and ORF8b (84aa), with 10% and 16% protein homology with SARS-CoV-2 ORF8, respectively [22,56]. While quickly dominating the SARS-CoV landscape, this deletion was followed by additional genomic deletions of up to 415 nucleotides which eventually resulted in the complete loss of ORF8 in later epidemic stages [57]. Although it remains to be determined whether SARS-CoV-2 will also eventually lose ORF8, an active role of ORF8 polymorphisms is anticipated in the progression of COVID-19 into an endemic disease.

The contribution of ORF8 to SARS-CoV-2 pathogenicity is further supported by several in vitro and in vivo studies of COVID-19 utilizing Δ*ORF8* SARS-CoV-2 mutants. For instance, several groups have revealed that Δ*ORF8* SARS-CoV-2 had reduced replication capabilities in immune-competent human pulmonary cells, particularly in angiotensin-converting enzyme 2 (ACE2)-expressing A549 (hACE2-A549) cells and in induced human pluripotent stem cell-derived lung alveolar type II (iAT2) cells, and it generated fewer and less infectious viral particles than wild-type SARS-CoV-2 in viral plaque assays [58,59,60]. Consistent with findings in COVID-19 patients, Syrian hamsters infected with Δ*ORF8* SARS-CoV-2 experienced milder disease, with reduced immune infiltration, lung damage, and inflammatory cytokine production compared to wild-type, despite no significant effect on viral titer [61]. Detailed studies in K18-human ACE2 transgenic mice did not show a significant impact of *ORF8*-deletions on SARS-CoV-2 morbidity, mortality, viral lung titers, inflammation, and tissue damage [58,60]. While these confounding results can be partly attributed to the SARS-CoV-2 strains used and the specific ORF8 mutants generated, they may also arise from the inter-species differences which may undermine the immunomodulatory activity of ORF8 in animal models. For instance, ORF8 has been demonstrated to bind and stimulate hyper-inflammation from the human IL-17 receptor (to be discussed in later sections), but it may not exert the same effect on the murine counterpart [7,46]. Overall, the characterization of Δ*ORF8* variants reveals the importance of ORF8 in establishing SARS-CoV-2 infection and suggests its significant role in both viral replication and immune dysregulation.

## 4. ORF8 Contributes to Viral Replication by Modulating Cellular Pathways and Gene Expression

Coronavirus accessory proteins are not typically implicated in replication directly, yet they commonly assist in creating an ideal host environment for the virus to proliferate [27]. Likewise, a plethora of SARS-CoV-2 ORF8 functions can be better understood by examining how they reprogram the host cell at a molecular level and facilitate effective viral replication. 

### 4.1. Induction of ER Stress

**Figure 2:**
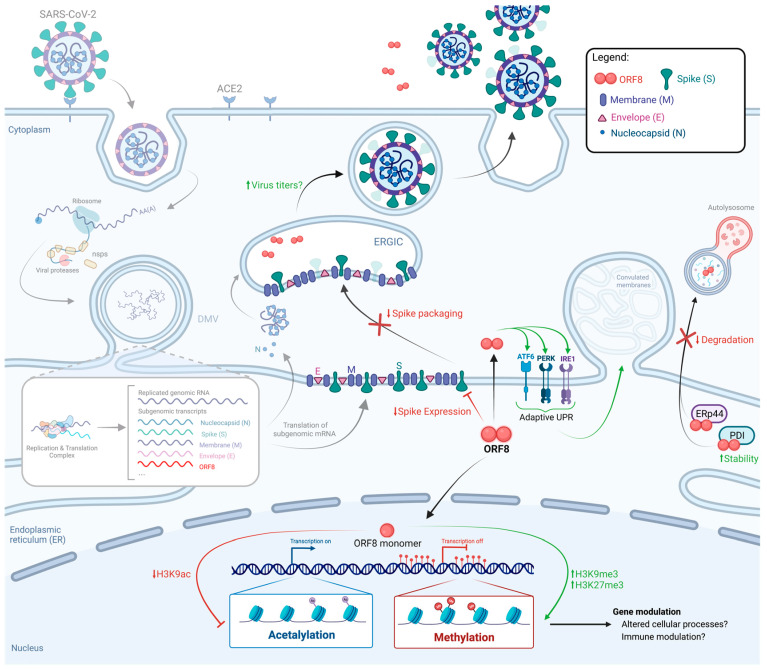
Modulation of cellular pathways promoting viral replication by SARS-CoV-2 ORF8. Replication cycle of SARS-CoV-2 is illustrated from virus entry, viral RNA replication, and assembly, to virus particle release. Viral and cellular events known to be modulated by ORF8 include ER stress responses, histone post-translation modifications, and spike expression. Reduced spike expression by ORF8 is represented as dimmed spike protein. Processes modulated by ORF8 are highlighted in high contrast, while steps of the SARS-CoV-2 life cycle unaffected by ORF8 (entry, replication, and translation) have been dimmed. M, Membrane; E, Envelope; N, Nucleocapsid; ER, Endoplasmic reticulum; ERGIC, ER intermediate compartment. Created in BioRender.com, accessed on 9 March 2023.

Altered cellular processes, which are commonly induced during viral infection, can promote the accumulation of unfolded and misfolded proteins leading to ER stress (ERS) [62]. To restore homeostasis, ERS induces the activation of the unfolded protein response (UPR), which can either result in terminal (apoptotic) UPR or lead to adaptive changes which contribute to cell survival [62,63]. In the context of SARS-CoV-2 infection, intracellular ORF8 forms mixed disulfide bonds and highjacks ER resident proteins involved in the secretory pathway, such as PDI and eRp44, to increase its stability and avoid degradation (Figure 2) [64]. Additionally, the binding of ORF8 to ER chaperones, such as BiP and calnexin, and to the three main ER stress sensors (inositol-requiring transmembrane kinase endoribonuclease-1a, IRE1a; protein kinase R-like endoplasmic reticulum kinase, PERK; and activating transcription factor 6, ATF6), induces the activation of adaptive UPR, rather than terminal UPR, thus preventing apoptosis (Figure 2) [7,64,65]. Activation of adaptive UPR by SARS-CoV-2 ORF8 leads to ER remodeling through the formation of convoluted membranes (CM) in transfected HEK293T cells (Figure 2) [64]. These findings highlight the potential of ORF8 in aiding SARS-CoV-2 assembly, as ORF8 directly increases cargo protein export from the ER, and UPR activation supports the release of progeny virions [64,65]. This feature of ORF8 may not be unique to SARS-CoV-2, since SARS-CoV ORF8ab also promotes the induction of ATF6 signaling to facilitate protein folding and processing [20,65].

### 4.2. ORF8 as a Histone Mimic

ORF8 further interferes with host cell functions by disrupting the transcriptional response to SARS-CoV-2 through histone H3 mimicry. Cellular gene expression is tightly regulated by epigenetic modifications on histone proteins, including histone H3 [59]. Within histone H3, one of the most critical regulatory sites is the ‘ARKS’ sequence within its two ARKSAP motifs [59]. These motifs undergo acetylation and methylation on lysine 9 and 27, respectively, associated with gene activation and repression [59]. Important to its function, SARS-CoV-2 ORF8 contains this ARKSAP motif between amino acid positions 50 to 55, which is not present in SARS-CoV ORF8ab, ORF8a, or ORF8b [59,66,67]. In an ARKSAP-dependent manner, ORF8 associates with histone H3, nuclear lamin-complex proteins, and the histone acyltransferase KAT2A, which it downregulates [59]. These ORF8-mediated interactions modulate histone post-translational modifications to promote chromatin compaction, inducing the trimethylation of histone H3 on lysine 9 (H3K9me3) and 27 (H3K27me3), while preventing H3 acetylation on lysine 9 (H3K9ac) (Figure 2) [59]. As a result, infection of human lung cells (A549) with Δ*ORF8* SARS-CoV-2 showed attenuated viral replication and reduced antagonization of epigenetic modifications [59]. 

Histone mimicry is not novel to SARS-CoV-2. Several other viruses, including human herpesviruses, adenoviruses, hepatitis C viruses, and influenza A virus, encode proteins containing ARKS or ARKT motifs, facilitating transcriptional modulation and the suppression of host cell antiviral responses [68]. Many of these proteins are localized to the nucleus, unlike SARS-CoV-2 ORF8, which has been reported to be localized in the cytoplasm with a strong ER association [6,34,64,68,69]. Interestingly, while ORF8′s novel capacity to dimerize is thought to contribute to its immunomodulatory functions, acetylation of K53 within its ARKS motif would likely prevent dimerization, suggesting the potential of monomeric ORF8 as a histone mimic [59]. However, the specific pathways targeted by ORF8-mediated epigenetic dysregulation remain undetermined. Identification of these pathways may reveal the cellular processes that ORF8 promotes to enhance viral replication.

### 4.3. Modulation of Spike Expression and Packaging

ORF8 also appears to affect the final step of the SARS-CoV-2 replication cycle, namely, the assembly and release of progeny virions. ORF8 has been shown to directly target the SARS-CoV-2 S protein for degradation, antagonizing S packaging into pseudoviruses, leading to reduced expression and cleavage of the S protein at the cell surface (Figure 2) [38,70]. Although the downregulation of the S protein could hinder SARS-CoV-2 transmission, it may also serve as a mechanism against immunosurveillance through reduced cell-surface S protein expression on infected cells. Furthermore, increasing the levels of S protein has been shown to lower viral titers in a SARS-CoV-2 virus-like particle (VLP) model expressing the structural proteins of SARS-CoV-2, suggesting that an optimal amount of S protein is required to support efficient virus production and ORF8 may provide such a viral mechanism [71,72,73]. However, the emergence of both S and ORF8 mutations decreasing ORF8′s antagonization of S protein expression indicates that SARS-CoV-2 may have adapted to exploit ORF8′s immunomodulatory functions while maximizing viral fitness [70].

## 5. ORF8 Modulates Host Immunity to Promote Immune Evasion

During SARS-CoV-2 infection, host immune responses are critical for mitigating viral establishment and disease severity. Upon target cell entry, innate immune sensors are activated to trigger the expression of type I interferons (IFNs), subsequently resulting in interferon stimulated genes (ISGs) and pro-inflammatory cytokine production [74]. Viral detection also induces downstream T and B cell activation, leading to the production of antibodies and mature cytotoxic CD8+ T cells [74]. SARS-CoV-2 has therefore been pressured to evolve an arsenal of strategies for host immune evasion, in which the role of ORF8 as an immunomodulator at the cellular and systemic level is significant. 

### 5.1. Antagonization of the IFN Response

**Figure 3:**
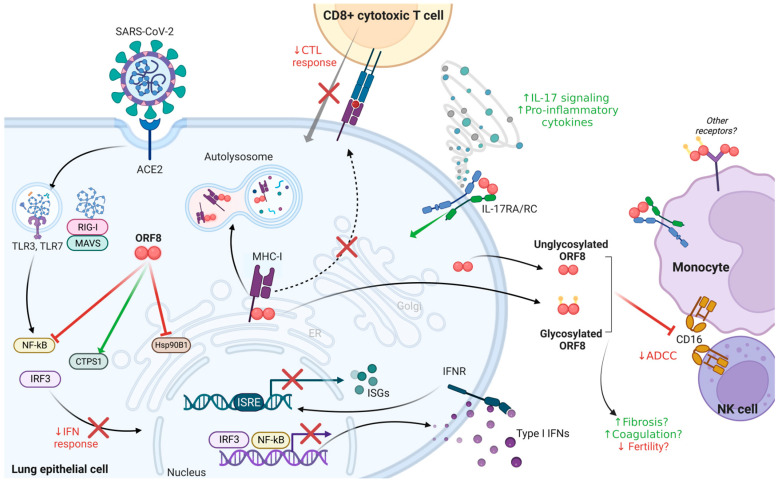
Modulation of host immunity by SARS-CoV-2 ORF8. ORF8 modulates the type-I IFN response, MHC-I antigen presentation and the CTL response, IL-17 signaling, and monocyte functions such as ADCC. Created in BioRender.com, accessed on 9 March 2023.

Type I IFN production and signaling is central to antiviral responses, with viral sensing by TLRs 3/7 or RIG-I/MDA5 triggering IRF3- and NF-kB-dependent transcriptional expression of type-I IFNs such as IFN-β, resulting in autocrine or paracrine induction of ISGs via interferon stimulated elements (ISREs) [75]. In HeLa (cervical cancer) and HEK293T (kidney) epithelial cells, ORF8 is capable of inhibiting mRNA expression of critical antiviral defenses such as IFN-β, NF-kB, and various other ISGs [10,76] (Figure 3). ORF8 also inhibits the basal-level expression of antiviral effectors, such as 2′,5′-oligodenylate synthetase 3 (OAS3) and IFN-induced transmembrane 1 (IFITM1) protein, thus inhibiting intrinsic immunity-associated molecules in addition to disrupting IFN-signaling [77]. Several molecular mechanisms have been reported for ORF8 to antagonize IFN signaling pathways (Figure 3). For instance, ORF8 directly prevents an efficient cytosolic dsRNA-sensing response downstream of RIG-I/MDA-5-MAVS by binding and activating CTP synthase 1 (CTPS1), leading to decreased IRF3 nuclear translocation [10,78,79]. Although downregulation of CTPS1 has been shown to promote SARS-CoV-2 replication and may impair T cell-mediated immunity, the specific mechanism and whether this antagonization is unique to ORF8 remains to be elucidated [79]. Additionally, ORF8 targets the ER chaperone heat shock protein 90 β family member 1 (HSP90β1) to inhibit its expression, leading to antagonization of the IFN-I pathway and decreased IFN-I related cytokine expression [10]. Supporting the role of ORF8 as an IFN antagonist, the T cell receptor (TCR) and HSP90β1 have been predicted to be upregulated in patients infected with the Δ382 *ORF8* variant, leading to increased cellular stress and immune responses [50]. Intriguingly, ORF8 was not found to decrease IFN-β-mediated immunity in a lung cell line (A549) but rather forms cytosolic and nuclear aggregates which antagonize the IFN-γ pathway through the inhibition of antiviral effectors [77]. IFN antagonization is not unique to ORF8, nor to SARS-CoV-2, as both SARS-CoV ORF8b and ORF8ab interact with IRF3, promoting its ubiquitin proteasome-dependent degradation and inhibiting IFN induction [20,80]. In addition, SARS-CoV-2 proteins, including N, ORF3b, ORF6, ORF7a, and ORF7b, have also been shown to be capable of downregulating the IFN-I response, with N and ORF6 being more potent antagonists than ORF8 [10,27,76,81]. The relative contribution of ORF8 to viral IFN evasion has yet to be fully elucidated. While ORF8 antagonization of this antiviral pathway may not be its primary role compared to other SARS-CoV-2 factors, its ability to assist in immunomodulation via this mechanism likely contributes to viral pathogenesis [10,76,77,81]. 

### 5.2. Downregulation of MHC-I to Counter the CTL Response

The major histocompatibility complex class I (MHC-I) is ubiquitously expressed on nucleated mammalian cells and is essential for cytotoxic T lymphocyte (CTL or CD8+) killing of virus-infected cells via antigen presentation and recognition (Figure 3) [82]. COVID-19 patients have been reported to suffer from CD8+ lymphocyte dysfunction, and examination of nasopharyngeal swabs of SARS-CoV-2 infected patients has revealed a mean reduction of 66% in MHC-I expression when compared to healthy patients [11,83]. These data indicate a potential viral mechanism of antigen presentation antagonization by SARS-CoV-2. Concordantly, an early study showed that ORF8 downregulates MHC-I by binding and targeting this immune receptor for lysosomal degradation, as a result protecting SARS-CoV-2-infected cells from CTL killing [11], which was corroborated by other reports [34,70]. In silico analysis further predicted that the main sites in ORF8 which mediate its binding to MHC-I involve positions 39–42 (IHFY), 104–107 (FYED), and 110–112 (EYH), while the deletion of positions 119–120 in the ORF8 of SARS-CoV-2 variant Delta decreases binding compared to wild-type ORF8 [84]. Downregulation of antigen presentation by SARS-CoV-2 was further expanded on by studies from other groups demonstrating that ORF3a, ORF6, and ORF7a are also potent viral antagonists of MHC-I signaling [83,85,86]. Interestingly, two of these groups did not observe ORF8 downregulation of MHC-I, suggesting that this activity of ORF8 might be dependent on specific cellular conditions which require additional investigation [83,86]. Overall, these findings highlight the need for SARS-CoV-2 to counter the CTL response via MHC-I downregulation by multiple mechanisms [83,85,86]. Interestingly, SARS-CoV ORF8ab, ORF8a, and ORF8b do not antagonize MHC-I, suggesting this function is specific to SARS-CoV-2 ORF8 [11,16]. 

### 5.3. ORF8 as a Virokine

In addition to its intracellular immunomodulatory properties described above, ORF8 also acts as a virokine to modulate immunity upon its secretion. The release of virokines, or virally encoded proteins imitating host cytokines, is a common immunomodulatory strategy utilized by several viruses, including poxviruses and herpesviruses [87]. Prior to the COVID-19 outbreak, it was reported that a feline coronavirus, feline infectious peritonitis virus (FIPV), secretes its accessory protein 7b during infection which likely acts as an immune modulator [88]. Concordantly, immunological mimicry through ORF8 secretion is an additional mechanism employed by SARS-CoV-2 to promote its pathogenesis. 

As a member of the Ig domain superfamily, ORF8 exhibits structural mimicry of immune molecules via its Ig domain, of which its imitation of interleukin 17A (IL-17A) has been demonstrated (Figure 3) [7,46]. As a pro-inflammatory cytokine, IL-17A signaling has been related to the hyper-inflammatory state and lung inflammation observed in severe COVID-19, with disease severity positively correlating with IL-17A levels [49,89]. Mimicking IL-17A, ORF8 signals through the IL-17A receptor (IL-17R) family by binding the extracellular fnIII-D2 domain of IL-17RA and inducing its heterodimerization with IL-17RC, producing a stronger and broader response than IL-17A itself [7,46]. In IL-17A-deficient mouse models, ORF8-induced secretion of pro-inflammatory factors (IL-6, IL-1β, TNF-α, IL-12) and consequent lung inflammation was blocked with an IL-17RA neutralizing antibody [7]. Additionally, ORF8 treatment of monocytes, which not only express high levels of IL-17RA but strongly interact with ORF8 in vitro, also activated the IL-17A pathway and inflammatory cytokine expression (CCL10, CXCL1, CXCL2, IL-6) to a greater level than IL-17A, which IL-17RA neutralization similarly blocked [46]. Recent evidence suggests that unglycosylated, rather than glycosylated, ORF8 is responsible for this effect, although the impact of this in COVID-19 infection remains unclear given clinical evidence of ORF8 glycosylation in patient sera [35,36]. Still, ORF8′s ability to imitate IL-17A appears to contribute to immune system hyperactivation and cytokine storm associated with poor COVID-19 prognosis [35,46,90]. This is revealed further by the frequently emerged L84S ORF8 mutation leading to reduced IL-17RA binding on monocytes and attenuated inflammation in vitro [46]. It is therefore interesting to consider how this role of ORF8 may cooperate with its other immune evasion functions, namely, its inhibition of both IFN production and signaling, and cytotoxic T-cell responses. It is likely that the initial delay in IFN production and MHC-I downregulation by ORF8 hinders the host’s antiviral response to benefit viral replication, and the subsequent rapid increase in pro-inflammatory cytokines and chemokines results in excessive immune infiltration causing tissue damage. Nevertheless, these anti- and pro-inflammatory functions of ORF8 contrast one another and add to the complexity of SARS-CoV-2 pathogenesis.

ORF8 interaction with IL-17RA is implicated in ORF8 binding to monocytes in human blood, as well as to both THP1 and U937 monocytic cell lines [35,36,41,46,61]. Concordantly with the fact that monocytes are major drivers of the atypical cytokine storm contributing to severe COVID-19, ORF8-treatment of either CD14+/CD16+ monocytes or THP1 cells also results in pro-inflammatory cytokine overexpression (Figure 3) [35,36,46,61,91,92]. Ongoing work reveals that ORF8-monocyte interactions may not influence the differentiation of these cells into dendritic cells (DCs), but rather promote DC maturation and resulting cytokine storm [41]. While the mechanism for the initial interaction of monocytes with SARS-CoV-2 ORF8 remains poorly defined, it has been revealed that CD14+ monocytes are the primary targets for ORF8 binding in whole blood samples, and that this binding is partly mediated through IL-17RA [46]. Correspondingly, it has been demonstrated that the intercellular communication between epithelial and monocytic cells, which results in cytokine release in COVID-19, is interrupted through either infection with a Δ*ORF8* SARS-CoV-2 virus or use of IL-17RA deficient monocytes [35]. Given ubiquitous expression of IL-17RA on different immune cells including T cells, B cells, and neutrophils which ORF8 does not interact with, additional host factors might contribute to ORF8-monocyte binding. For example, we have found that ORF8 interacts with another Ig domain superfamily member, Fcγ receptor IIIa (FcγRIIIa; CD16a), allowing ORF8 to bind CD14+CD16+ monocytes, and to a lesser extent NK cells, in vitro (Figure 3) [9]. Soluble ORF8 also decreases CD16a levels on the surface of both cell types dependently of its interaction with monocytes, although it is unclear whether this occurs through ORF8-mediated CD16a endocytosis or surface shedding [9]. Consequently, antibody-dependent cellular cytotoxic (ADCC) responses mediated by plasma from convalescent COVID-19 patients and vaccinated individuals were decreased in the presence of ORF8 [9]. Together with its downregulation of MHC-I to prevent the elimination of virally infected cells, this impact on CD16a by ORF8 further contributes to SARS-CoV-2 evasion of the humoral response [9]. It remains to be determined whether the rapid evolution of ORF8 reflects a need for emerging SARS-CoV-2 variants to select for ORF8 mutations that are better able to evade antibody responses and their Fc-mediated functions. ORF8-monocyte interactions are made more elaborate by the observation of abortive infection of monocytes in COVID-19 patients, where intracellular ORF8 expression within these cells may further antagonize monocyte function [93].

### 5.4. Beyond Immunomodulation

Along with its important immunomodulatory role, secreted ORF8 has been implicated in several other clinical manifestations of COVID-19. For instance, pulmonary fibrosis and dysregulated coagulation have been observed in COVID-19 patients (Figure 3) [94,95]. An in silico phylogenetic analysis of ORF8-binding proteins and their interactors have revealed a cluster of proteins strongly associated with complement and coagulation cascades, as well as lung fibrosis [78,96]. Transcriptomic analysis of ORF8-treated monocytes has also suggested that ORF8 upregulates the expression of key factors involved in these pathways [46]. In support of these proteomic and transcriptomic analyses, SARS-CoV-2 ORF8 was reported to bind the β-chain of complement component C3b and, as a result, inhibit the complement amplification loop [97]. Moreover, an impact on fertility by SARS-CoV-2 has been reported by several groups, suggesting that the virus may cause edema of the testes, impair sperm motility and function, and damage seminiferous tubules [98,99,100]. The role of ORF8 in this disease outcome has been implicated, as ORF8 delivered to male mice of reproductive age resulted in decreased sperm motility, abnormal or loss of reproductive tissue architecture, and reduced fertility when mated (Figure 3) [37]. Although these results do not directly translate to human infection, the solubility of ORF8 in COVID-19 patient serum would allow this protein to reach distal sites such as the testes in human males. 

## 6. Conclusions

The unique properties of SARS-CoV-2 ORF8 summarized in this review are closely associated with viral pathogenesis and evolution. The hypervariable nature of ORF8 between SARS-CoV-2 variants and other Sarbecoviruses, as well as its newly acquired structural features, specifically its Ig domain and ability to dimerize, suggest an active role of ORF8 in the initial and continual adaptation of SARS-CoV-2 to human hosts. While carrying out several functions within infected cells including antagonization of ER stress, epigenetic regulation, viral spike expression, the IFN response, and CTL-mediated immunity, ORF8 also acts as a virokine to induce IL-17RA-mediated cytokine production and monocyte dysfunction. Overall, ORF8 makes a significant contribution to viral reshaping of the host at a cellular and systemic level, creating an environment in which viral production can efficiently occur in the presence of innate and adaptive antiviral immunity. Beyond these key functions, ORF8′s broad interactome indicates its additional roles in COVID-19, both intracellularly and extracellularly. It is also worth considering that ORF8 may interact and cooperate with other SARS-CoV-2 viral proteins. For instance, reports have shown that co-expression of ORF8 with either M, ORF3a, ORF7a, or E in vitro enhances cytokine induction by ORF8, supporting the notion that this accessory factor may additionally synergize with other viral proteins to cause severe disease pathology [36]. Given the high immunogenicity of ORF8 and the reduced disease severity in SARS-CoV-2 variants lacking this protein, ORF8 cannot be overlooked as a potential therapeutic target against COVID-19. However, both its hypervariability and systemic presence due to secretion present major limitations for the development of specific and long-lasting ORF8-based therapeutics. As a result, targeting host factors essential for ORF8 function could offer an alternate strategy. For example, ongoing work has successfully demonstrated that targeting CTPS1 can restore IFN production, thus diminishing SARS-CoV-2 replication [92,93]. Given the plethora of molecular and cellular mechanisms ORF8 has been shown to engage in, it will be highly interesting to measure the presence of this secreted viral protein in key anatomical sites and tissues in infected individuals, as well as investigate the potential impact of ORF8 on organ functions and its contribution to prominent clinical manifestations in COVID-19 patients.

## Data Availability

Data sharing not applicable.
